# Multipotential stromal cells in the talus and distal tibia in ankle osteoarthritis – Presence, potency and relationships to subchondral bone changes

**DOI:** 10.1111/jcmm.15993

**Published:** 2020-12-11

**Authors:** William G. Jones, Jehan J. El‐Jawhari, C. L. Brockett, Lekha Koria, Ioannis Ktistakis, Elena Jones

**Affiliations:** ^1^ Faculty of Medicine and Health Leeds Institute of Rheumatoid and Musculoskeletal Medicine University of Leeds Leeds UK; ^2^ School of Mechanical Engineering Institute of Medical and Biological Engineering University of Leeds Leeds UK; ^3^ Department of Biosciences School of Science and Technology Nottingham Trent University Nottingham UK; ^4^ Clinical Pathology Department Mansoura University Mansoura Egypt; ^5^ Hull and East Yorkshire Teaching Hospital Hull UK

**Keywords:** ankle, multipotential stromal cells, osteoarthritis, regenerative medicine

## Abstract

A large proportion of ankle osteoarthritis (OA) has an early onset and is post‐traumatic. Surgical interventions have low patient satisfaction and relatively poor clinical outcome, whereas joint‐preserving treatments, which rely on endogenous multipotential stromal cells (MSCs), result in suboptimal repair. This study investigates MSC presence and potency in OA‐affected talocrural osteochondral tissue. Bone volume fraction (BV/TV) changes for the loading region trabecular volume and subchondral bone plate (SBP) thickness in OA compared with healthy tissue were investigated using microcomputed tomography. CD271‐positive MSC topography was related to bone and cartilage damage in OA tissue, and in vitro MSC potency was compared with control healthy iliac crest (IC) MSCs. A 1.3‐ to 2.5‐fold SBP thickening was found in both OA talus and tibia, whereas BV/TV changes were depth‐dependent. MSCs were abundant in OA talus and tibia, with similar colony characteristics. Tibial and talar MSCs were tripotential, but talar MSCs had 10‐fold lower adipogenesis and twofold higher chondrogenesis than IC MSCs (*P* = .01 for both). Cartilage damage in both OA tibia and talus correlated with SBP thickening and CD271+ MSCs was 1.4‐ to twofold more concentrated near the SBP. This work shows multipotential MSCs are present in OA talocrural subchondral bone, with their topography suggesting ongoing involvement in SBP thickening. Potentially, biomechanical stimulation could augment the chondrogenic differentiation of MSCs for joint‐preserving treatments.

## 1 INTRODUCTION

Osteoarthritis (OA) has an estimated prevalence of 10%‐30% worldwide^1^, ^2^; however, most interventions treat the symptoms of the disease, rather than the cause. Ankle OA creates a particular problem, as the disease is estimated to be post‐traumatic in anywhere between 18% and 75% of cases.^3^, ^4^, ^5^ This results in a much earlier age of onset, with the average symptomatic ankle OA patient being aged only 51.5 years, compared with 63 in the hip.^5^, ^6^, ^7^ The structure of the ankle is different from the knee or hip, with far more congruency, thought to be related to the entire joint being used to offset load, rather than support soft tissue.^8^ There is a dire need to understand how this affects OA, and how this relates to the cellular level, in order to enhance the efficacy of regenerative repair treatments through evidence‐based design.

The current treatment with the best clinical outcome is the fusion of the ankle, which is typically employed at age 68 years, approximately 16 years after onset.^5^ This waiting period creates a need for earlier, joint‐preserving interventions. Total ankle replacement is another surgical treatment option, having higher patient satisfaction; however, there is a shorter life‐time of the treatment.^9^ Early‐stage interventions are mostly analgesics, or attempts to induce natural regeneration using joint‐resident cells.^10^ Typical early‐stage interventions include the removal of damaged cartilage, usually followed with further regenerative treatment. One example is microfracture, which involves drilling into the bone until the release of fat and blood, simultaneously releasing bone marrow resident reparative cells to create de novo cartilage. Other options include autologous osteochondral transplant (AOT) or joint distraction.^11^, ^12^ Each of these treatments relies on endogenous cells to remodel the nearby area.^13^ These treatments show high success rates and patient satisfaction up until 2 years; however, the formation of fibrocartilage eventually leads to treatment failure.^14^

Local multipotential stromal cells (MSCs) are implicated in these treatments as the major regenerative cell within the joint; however, their behaviour is poorly understood within the ankle compared with other joints like hip and knee.^13^ As MSCs are unlikely to be transported by blood^15^, ^16^; the subchondral bone, synovial fluid or synovium are considered to be the main MSC tissue sources within joints.^17^ In ankle OA, MSCs have been isolated from the synovial fluid of patients; however, MSC presence in talocrural subchondral bone, from which reparative cells released by microfracture would come from, remains unexplored.^18^ No existing study compares resident subchondral bone MSC topography and differentiation capacity between talus and distal tibia in ankle OA patients. A clear understanding of resident MSCs behaviour, and how tissue damage affects their function, from both biological and mechanical standpoints, is of major importance for the development of novel regenerative medicine strategies to treat ankle OA or osteochondral lesions.^19^

This study utilizes osteochondral talocrural specimens excised during ankle fusion to investigate the relative abundance and differentiation capacity of MSCs in talar and distal tibia subchondral bone. It also aims to analyse MSC tissue topography in relation to cartilage damage and bone sclerosis in these specimens. We show that MSCs with an osteochondral‐reparative phenotype are present in ankle OA joint and, notably localize near the OA joint surface, however, are highly correlated with regions of bone sclerosis, as such may be key drivers. These findings will help to inform future developments of enhanced regenerative therapies, such as microfracture, to enable an optimal release of MSC from the talar bone.

## 2 METHODS

### 2.1 Patient samples

Ethical approval for human ankle tissue collection (07/Q1205/27) and control Iliac crest (IC) tissue collection (06/Q1296/127) was obtained from NREC Yorkshire and Humberside National Research Ethics Committee, in compliance with the Helsinki Declaration of ethical principles for medical research involving human subjects. For these experiments, seven ankle OA patients (median age 66 years, range 34‐73, 4 male 3 female) were included after signed consent was received, whether or not OA was post‐traumatic was not known. Each patient underwent fusion of the talocrural joint for OA and was ambulatory prior to surgery. IC bone from 3 donors was obtained from patients undergoing orthopaedic surgery for metal removal following previous fracture, who were otherwise healthy (ages 32, 38 and 42, 2 male, 1 female). Samples were placed immediately into saline after surgery and transferred to the laboratory. Non‐diseased human cadaveric ankles (age 40‐60 years, all‐male, no evidence of ankle OA) were obtained from Medcure, USA, under local university ethics, and stored at −80°C before distal tibia and talus were removed for imaging. Freezing meant that cadaveric samples were unsuitable for subsequent cell analysis.

### 2.2 Microcomputed tomography (mCT) and image analysis

Three pairs of ankle OA talar and distal tibial osteochondral samples (approximately 35 mm × 25 mm × 9 mm and 28 mm × 2 mm × 7.8 mm from tibia and talus respectively) were retrieved and stored at −80°C. Following being defrosted and submerged in PBA, samples were imaged using mCT in a SkyScan 1278 (Bruker), at an 18‐μm isotropic resolution. The cadaveric, non‐diseased ankle bones were scanned at a 16‐μm isotropic resolution using a micro‐CT 100 (ScanCo Medical). Anatomic areas were matched between the talus and tibia, selecting regions of loading. Bone volume of total volume (BV/TV) and subchondral bone plate (SBP) thickness were measured according to standard protocols. Briefly, BV/TV was measured using the BoneJ plug‐in for NIH ImageJ (US National Institutes of Health), up to 3 mm depth from the SBP.^20^, ^21^, ^22^ SBP thickness was measured by reorientating the image to a transverse view and measuring the perpendicular distance from the beginning of bone to the first visible trabecular gap. Measurements were taken at twenty points for each bone and averaged.

### 2.3 Histology and immunohistochemistry

Talus and distal tibial sections from 4 ankle OA patients were fixed in 3.7% formaldehyde (Sigma) for 1 week and subsequently decalcified in 0.5 mol/L EDTA (Fisher Scientific Ltd) for at least 3 months, using dental X‐ray (CS2200, Carestream Dental) to confirm decalcification. Once decalcified, samples were fixed for a further 48 hours in 3.7% formaldehyde and then embedded in paraffin blocks.

Safranin O staining was performed on 5‐µm sections of whole tibias and proximal tali according to standard protocols (all reagents from Sigma). Safranin O stained slides were scanned at 10× using the Leica Aperio T2 (Leica Biosystems), and images were taken using Aperio Imagescope v12.4.0 (Leica Biosystems). Cartilage damage across the whole sagittal plane of both the talus and distal tibia was assessed by two independent observers using the OARSI OA cartilage histopathology system.^23^ Talus and tibial sections were additionally analysed using ImageJ v1.52a.^20^ Firstly, the SBP thickness was measured as previously described.^24^, ^25^ Briefly, the distance from the tidemark of calcified cartilage to the subchondral bone space was measured. The ratio of bone area to total area (BA/TA), starting beneath the SBP, was calculated across the whole joint by splitting the joint into series of tiles, up until 3 mm bone depth.

Immunohistochemistry for CD271‐positive cells, a broadly used marker of in vivo bone‐resident MSCs,^26^, ^27^ was performed with monoclonal mouse anti‐human antibody (clone ME20.4; Invitrogen) at 1:200 dilution followed by incubation with the EnVision + Dual Link System‐HRP including horseradish peroxidase and 3,3′‐diaminobenzidine tetrahydrochloride (DAB) (Dako, Agilent) as previously described.^28^ Images were taken on a Nikon Eclipse Ti2‐E camera (Nikon) and analysed using the Nuance Multispectral Imaging System (Caliper Lifesciences) for the bone area and CD271‐positive area.^29^

### 2.4 MSC isolation and culture

Talus and distal tibia samples of 3 ankle OA patients were submerged in phosphate buffered saline ((PBS) Thermofisher), and articular cartilage and soft tissue were removed using a scalpel and subsequently discarded. Remaining subchondral bone was minced mechanically into small fragments using a rongeur and then weighed. Samples were digested for 4 hours at 37°C in 3000 units of collagenase (Worthington Biochem) per gram of bone, with agitation every 30 minutes. The liquid fraction was filtered through a 44‐µm pore filter (Corning). The filtrate was then passed through a 22‐µm pore filter before centrifugation at 300 *g* for 10 minutes to pellet extracted cells.^30^ Cells were counted using haemocytometer and used for CFU‐F assay or culture expansion. For culture expansion, 10^6^ cells were plated per 25‐cm^2^ flask (Corning) in StemMACS™ MSC Expansion Media (Miltenyi Biotec) supplemented with 1% penicillin/streptomycin (P/S, Thermofisher). Media was changed twice per week. Cells were split at 80% confluency using trypsin (Thermofisher). Subsequent passages were plated at 10^5^ MSCs per flask. MSCs were grown to either passage 2 for surface marker expression measurement by flow cytometry, or passage 3 for trilineage differentiation. The IC MSCs were isolated and expanded with the same method except that the removal of cartilage and soft tissue was not necessary. Non‐diseased human ankles could not have cells extracted as freezing of tissue made them unsuitable for downstream analyses.

### 2.5 CFU‐F assay

MSCs were enumerated by CFU‐F assay; freshly isolated cells were seeded at densities 2.5 × 10^3^, 5 × 10^3^ and 1 × 10^4^ per dish in 600‐mm culture dishes (Corning) in duplicate with StemMACS expansion media supplemented with P/S. Media was changed twice a week. Dishes were fixed 3.7% in formaldehyde at 14 days and stained with methylene blue to visualize colonies as previously described.^31^ Colony numbers were counted, averaged, and converted into CFU‐F/10^6^ cells and used to compare between talus, tibia and IC. Colony area and integrated density were measured using ImageJ. Briefly, colonies were converted to greyscale, and colony number and density were measured by the ‘analyse particles’ function in the program.^32^

### 2.6 Flow cytometry

Surface expression of culture‐expanded MSCs was assessed using the ISCT criteria of cultured MSCs.^33^ Briefly, after trypsinization, cells were pelleted by centrifugation at 500 *g* for 10 minutes, stained with relevant antibodies (all from Miltenyi Biotec) in the dark at 4°C for 15 minutes (positive markers: CD73‐PE (Clone AD2,), CD90‐PerCP‐Vio700 (Clone REA897), CD105‐FITC (Clone 43A4E1); negative markers (all VioGreen): CD14 (Clone REA599), CD19 (Clone LT19) CD34 (Clone AC136), CD45 (Clone REA747), HLA‐DR (Clone REA805)). Data acquisition was conducted using the Attune 2 Laser System (Invitrogen) and analysed using the Attune Cytometric Software (v2.1.0). Gates were applied to discount debris and then to measure the percentage of cells expressing these surface markers.

### 2.7 Trilineage differentiation

MSCs were induced into chondrogenic, adipogenic and osteogenic cell lineages using specific differentiation media (Miltenyi Biotec).^30^ Chondrogenesis was performed with 2.5 × 10^5^ MSCs seeded in quadruplicate 1.5‐mL Eppendorf tubes with 0.5 mL ChondroDIFF. For adipogenic differentiation, cells were cultured in triplicates in 1 mL AdipoDIFF for 21 days, at a density of 4 × 10^4^ per well in a 48‐well plate for Nile red/DAPI staining and 5 × 10^4^ per well in a 24‐well plate for oil red staining. Osteogenic differentiation was performed in triplicates with 1 × 10^4^ cultured MSCs per well in a 12‐well plate in 2 mL OsteoDIFF and cultured for 14 days for alkaline phosphatase and 21 days for Alizarin Red, calcium deposition and DNA measurements. Glycosaminoglycan (GAG) production was used to quantify chondrogenesis as previously described.^28^ Adipogenesis was quantified by measuring oil red‐positive area of the total area using ImageJ v1.52a. Osteogenesis was quantified using calcium deposition normalized to DNA content.^34^

### 2.8 Statistical methods

Statistics were performed with GraphPad Prism v7.04 (GraphPad). Wilcoxon signed‐rank test was used for mCT data, and Kruskal‐Wallis test with Dunn's post hoc test for multiple comparisons was used for all other tests. Significance was set at *P* < .05 (*P < .05, **P < .01, ***P < .001). The details of individual tests are presented in the respective figure legends.

## 3 RESULTS

### 3.1 Subchondral bone changes in the talus and distal tibia of ankle OA patients

**Figure 1:**
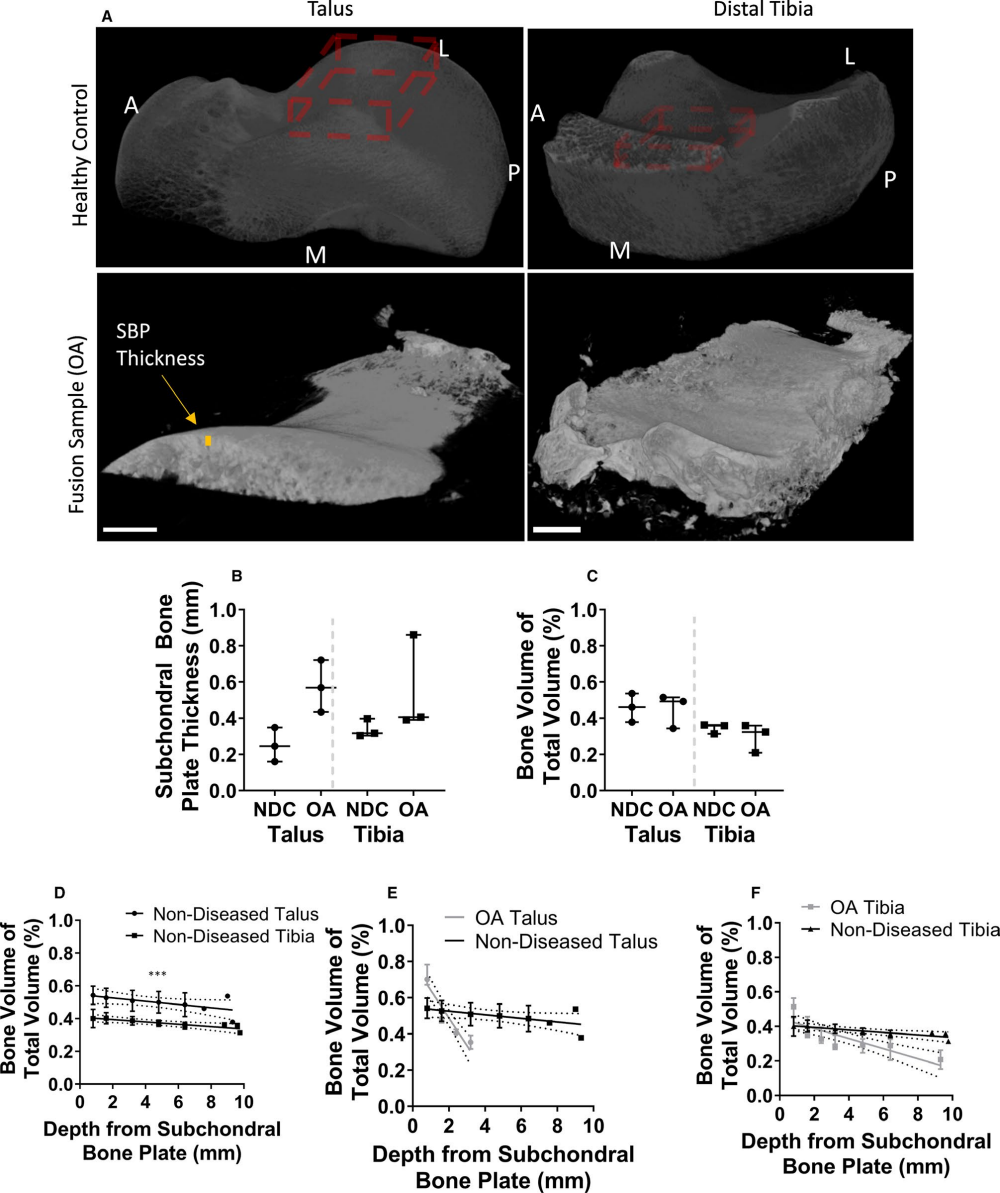
Morphological changes of OA visible by microcomputed tomography on proximal talus and distal tibia (n = 3 OA patients compared with n = 3 non‐diseased controls, NDC). A, Talocrural tissues rendered in 3D. Healthy control images approximate analysed regions. A—anterior, P—posterior, M—medial, L—lateral. The OA talus and tibia are far smaller due to the minimal amount of bone removed from surgery. B, Change in subchondral bone plate (SBP) thickness in OA (symbols represent an average 20 measurements per donor). C, Change in bone volume of total volume (BV/TV) in the analysed regions in OA talus/tibia (symbols represent individual donors). D, Change in BV/TV by depth in non‐diseased control talus/tibia. E, Change in BV/TV by depth from the SBP in talus. OA talus samples are measured to 4 mm depth due to thinner samples being removed during surgery. F, Change in BV/TV by depth from SBP in tibia. Symbols and error bars on B/C show medians ± interquartile ranges (IQR) for 3 donors with a minimum of 200 slices. D‐F show medians ± interquartile ranges (IQR) for 3 donors with 100 slices per depth interval ***P < .001, using Wilcoxon signed‐rank test

To investigate specific subchondral bone behavioural changes in OA‐affected bones of the ankle, mCT was performed and compared with healthy control cadaveric samples from approximately same anatomic regions (Figure 1, red regions for controls). As seen, OA samples were smaller than control samples, due to tissue removed being minimized during surgery. The maximal depth of talus in particular was 3mm below the SBP. SBP thickness was measured as the distance to the first trabecular space (demonstrated in orange on Figure 1A).

Quantification of bone changes (Figure 1B) revealed an apparent SBP thickness increase in OA compared with healthy in both talus and distal tibia, consistent with prior research in the ankle.^35^ Interestingly, the overall volume as a fraction of total volume (BV/TV) was unchanged between health and OA, measured to 3 mm depth from the SBP (Figure 1C). The talus appeared to have higher BV/TV than tibia in both OA and health, consistent with prior work.^8^ To investigate whether BV/TV in OA changed by depth from the joint surface, samples were segmented in millimetre depths, and BV/TV measured for each 1 mm. In health, the tibia and talus measurements showed a similar gradual decrease in BV/TV as the depth increased, with the tibia being significantly thinner than the talus at each depth (*P* = .001; Figure 1D). In OA, where the talus could be only measured up to 3 mm depth from the SBP, it showed much greater BV/TV increases near the SBP than in the tibia (Figure 1E), but also a depth‐dependent loss in BV/TV which fell below that of the healthy talus at 2 mm. The tibia showed a similar trend (Figure 1F), while not as severe as the talus, but more so than the healthy control. These microstructural changes in OA talus and distal tibia indicated the predominant bone formation near the joint surface by the SBP thickening and up to 2 mm beneath it.

### 3.2 MSC frequency and colony characteristics in OA distal tibia and talar bone

**Figure 2:**
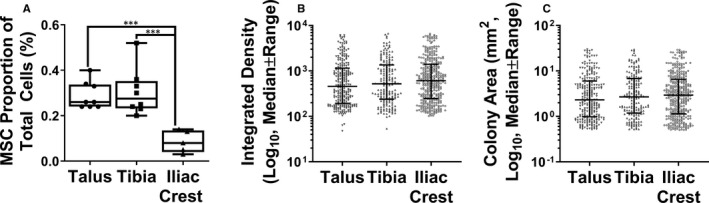
Properties of MSCs isolated from OA talus and tibia (n = 3 donors) and control Iliac crest (n = 3 donors) isolated by collagenase digestion of bone. A, Percentage of cells isolated which are MSCs, measured by CFU‐F assay. Graphs show median ± interquartile range for 3 repeated experiments from 3 donors. B, Integrated density of all MSC‐initiated colonies from CFU‐F assay measured using ImageJ. C, Area of all MSC‐initiated colonies from CFU‐F assay measured by ImageJ. Symbols on B and C represent individual colonies; lines and error bars represent medians and IQR. ***P < .001, Kruskal‐Wallis test with Dunn's correction for multiple comparisons

To investigate the presence and functionality of MSCs in OA talus and distal tibia, following collagenase extraction of all cells from bone, standard MSC enumeration and differentiation assays (donors ages 34, 58 and 66, 2 female and 1 male) were performed compared with control IC bone (donor ages 32, 38 and 4, 1 female and 2 male).^36^ Talus and distal tibia MSCs were found at a significant, 30‐fold higher frequency that in IC as a proportion of total cells ((Figure 2A), *P* = .0005, 0.0003, respectively). MSC colony‐forming behaviour was analysed by looking at the integrated density (Figure 2B) and colony area (Figure 2C). There were no apparent differences between talus, tibia or IC colonies reflecting similar in vitro MSC proliferative potential.

### 3.3 Differentiation capacity of MSCs from the OA talus and distal tibia

To confirm these colony‐forming cells were MSCs, plastic‐adherent cells were expanded in standard MSC conditions and their surface phenotype and differentiation capacity were measured using flow cytometry and standard trilineage differentiation assays, respectively.

**Figure 3:**
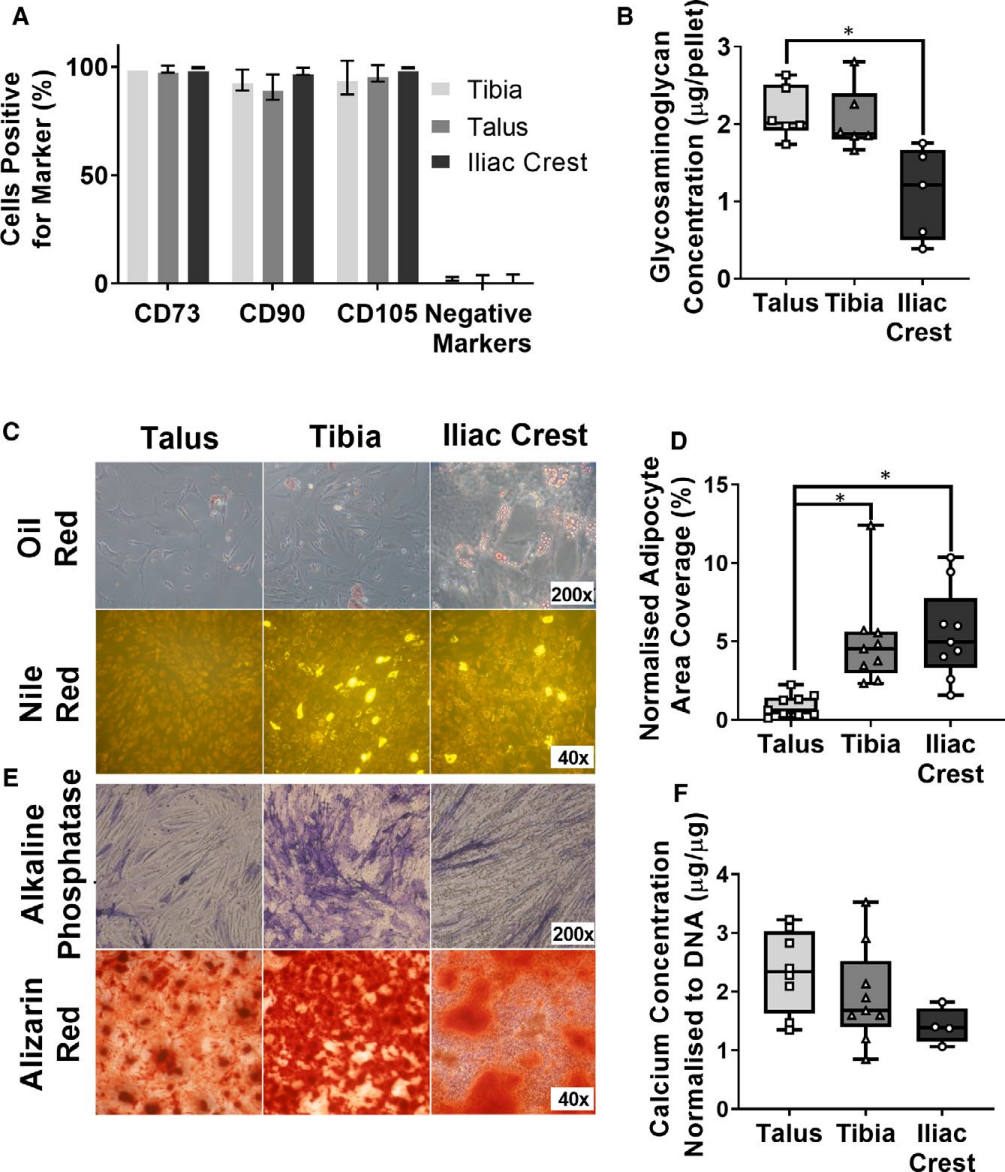
Characterization of culture‐expanded MSCs from OA distal tibia and talus (n = 3 donors) and control Iliac crest bone (n = 3 donors). A, Surface phenotype of isolated cells from talus, tibia and IC after culture to passage 2 by flow cytometry. Negative markers reflect absence of haemopoietic lineage cells. Bar graphs represent means and standard deviations (SDs). B, Glycosaminoglycan concentration of cartilage pellets following chondrogenic C/D adipogenic assays: C, Oil red and Nile red staining of adipocyte differentiated MSCs. D, Oil red‐positive area of total area. E/F Osteogenic assays: E, Alkaline phosphatase and Alizarin Red staining to prove osteogenic differentiation of MSCs. F, Calcium deposition of differentiated cells normalized to DNA content. Box plots represent medians and IQR for 3 repeated experiments from 3 donors. *P < .05, Kruskal‐Wallis test with Dunn's correction for multiple comparisons

Passage‐2 adherent cells from distal tibia, talus and control IC cells were 95% or more positive for CD73, CD90 and CD105 and less than 3% positive for CD14, CD19, CD34, CD45 and HLA‐DR (Figure 3A), consistent with MSC nature. All cultures successfully differentiated into bone, cartilage and fat lineages (Figure 3B‐F). GAG content in day‐21 chondrogenically differentiated pellets was similar between the OA talus and tibia; however, for each individual donor there was slightly higher GAG synthesis in the talus than tibia (Figure 3B). Both tibia and talus MSCs showed higher GAG concentration than the IC MSCs, although this difference was only significant for the talus (*P* = .012).

Adipogenic assays revealed that the talar cultures mostly showed the formation of ‘horseshoe’ fat‐laden structures, consisting of very small fat globules, suggesting slowed or halted differentiation (Figure 3C). Oil red staining area was significantly fivefold lower in talar cultures compared with OA tibia or control IC when normalized to total cell area (*P* = .024, .0005, respectively (Figure 3D)). Alkaline phosphatase activity at day 14 and Alizarin Red staining at day 21 were evident, suggesting mineralization and deposition of calcium (Figure 3E). OA talar MSCs trended noticeably higher calcium production compared with tibia MSCs, and particularly IC MSCs. However, this failed to reach statistical significance (Figure 3F). These results demonstrated that OA talus and tibial MSCs were tripotential, and capable of cartilage differentiation in vitro. Interestingly, the low level of OA talar MSC adipogenesis would suggest cells are primed for osteochondrogenesis.

### 3.4 Histological cartilage and bone changes in ankle OA

**Figure 4:**
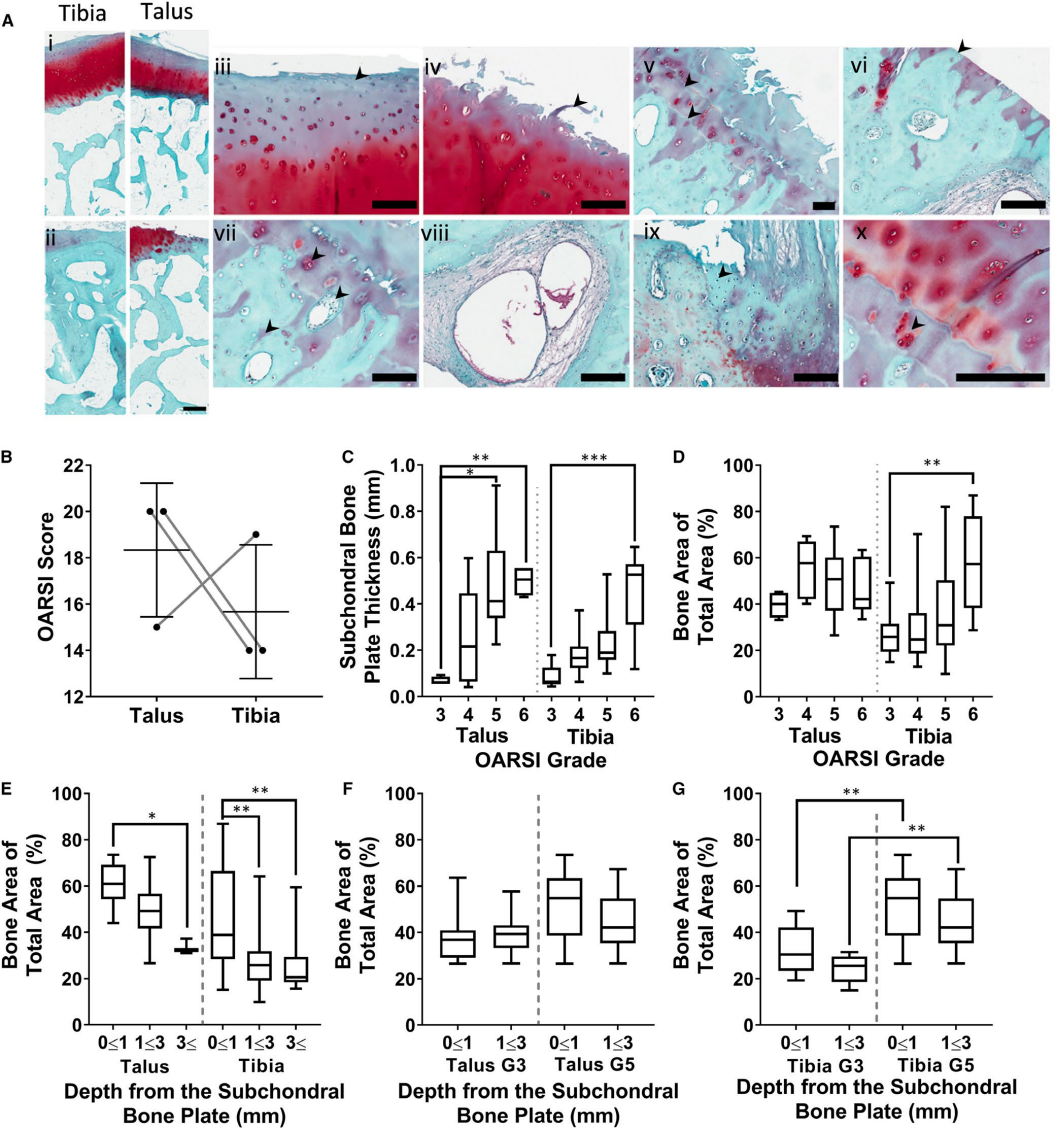
Ankle tissues morphology and cell topography in end‐stage osteoarthritis A, Safranin O histology of talus or distal tibial osteochondral tissue sections obtained from ankle fusion patients. Red staining shows glycosaminoglycans, and blue shows bone. (i—Examples of grade 3 distal tibia/talus. ii—Example of grade 5 distal tibia/talus. iii—Glycosaminoglycan loss in cartilage. iv—Fibrillation of cartilage. v—Fibrillation and tidemark duplication. vi—Denuded cartilage. vii—Chondrocyte clustering. viii—A subchondral bone cyst. ix—example of grade 6 tissue with osseous repair extending above the previous surface. x—and hypertrophy/clustering of chondrocytes (i scale bar 500 µm, ii‐ix scale bar of 200 µm). B, Comparisons in OARSI cartilage damage score between the tibia and talus. Symbols represent individual patients. C, Change in subchondral bone plate thickness (SBP) with cartilage damage grade. D, Bone area of total area beneath the subchondral bone plate (SBP) between the talus and tibia, compared with the OARSI grade of the overlying cartilage. E, Bone area of total area (BA/TA) comparing different bone regions with the distance from the SBP. F, Comparison of changes in BA/TA of talar bone in grade 3 vs grade 5 damaged regions near the subchondral bone plate vs further away. G, Comparison of changes in BA/TA of tibial bone in grade 3 vs grade 5 damaged regions near the subchondral bone plate vs further away. C‐G, Box plots represent medians and IQR from 3 donors measured from a minimum of 4 individual tiles. *P < .05, **P < .01, ***P < .001, Kruskal‐Wallis test with Dunn's correction for multiple comparisons

To investigate whether any chondral regeneration from these MSCs takes place in ankle OA, Safranin O staining of OA distal tibia and talus samples was performed and cartilage damage was assessed using standard OARSI scoring system.^37^ All samples presented characteristic OA osteochondral tissue morphology, although damage varied across the joint surface and between patients (Figure 4Ai representing early damage, ii representing severe damage). Less damaged areas presented GAG depletion (iii), progressing to minor (iv) and major cartilage fibrillation (v) whereas highly damaged regions presented complete cartilage denudation (vi) and extreme bone sclerosis (vii) (Figure 4A). Rarer abnormalities included subchondral bone cysts (viii) and osseous repair over the previous surface (ix). No significant differences in overall cartilage stage by OARSI score were found (Figure 4B) and no cartilage repair, seen as Safranin‐positive tissue spanning the lesion margins and in the vicinity of chondrocyte clustering,^38^ was evident.

To investigate whether bone formation correlated with regions requiring chondral repair, the pattern of cartilage damage was investigated in relation to the subchondral bone changes. Indeed, SBP thickness increased significantly (threefold to sevenfold) with increasing cartilage damage in both talus and tibia, similar to that seen in mCT (Figure 4C). In tibia, there was a similar relationship between cartilage damage and subchondral bone density, measured as bone area as a fraction of total area (BA/TA), but not so in the talus (Figure 4D).

BA/TA as a function of distance from the SBP was next compared between greatly damaged, grade 5 regions, to less damaged grade 3 regions. In both talus (Figure 4F) and tibia (Figure 4G), grade 5 regions displayed higher BA/TA at both 0‐1 and 1‐3 mm depths measured; however, these trends were more dramatic and statistically significant in tibia (Figure 4G), suggesting that sclerotic reaction to cartilage loss in the tibia may be stronger than in the talus.

Combined with mCT work, these data indicated that bone formation happens most near the joint surface, with increased SBP thickness and BV/TV closer to the joint surface. The bone response to cartilage loss may occur more rapidly in tibia than the talus, suggesting different biomechanical pressures on resident cells including resident MSCs.

To confirm recent bone formation in talocrural tissue samples, we utilized picrosirius red staining, which has been previously used for revealing the collagen fibre organization and maturity in bone.^39^, ^40^ This dye stains immature collagen in new bone red under biphasic light, and old, mature bone collagen yellow/green. New collagen fibres were seen aligned with the surface of the trabeculae, particularly in grade 5 OA tissues (Figure S1A). Quantitative measurements of red area revealed more prominent new collagen formation in tibia than the talus (Figure S1B). In both talus and tibia, grade 5 regions had a trend for higher proportions of red staining compared with grade 3 regions (Figure S1C), indicating more new bone formation in these damaged regions. In addition, new bone formation in talocrural tissues was evident by immunohistochemical staining for CD56 (Figure S2A), a molecule present on bone‐lining osteoblast progenitors, and E11, a marker of early embedding osteocytes, with both cell types present in the vicinity of cuboidal osteoblasts (Figure S2B). This illustrates the three progressive stages of the bone formation process in vivo.^29^, ^39^

### 3.5 Resident CD271+ MSC topography and relationship to bone changes in talus and distal tibia

**Figure 5:**
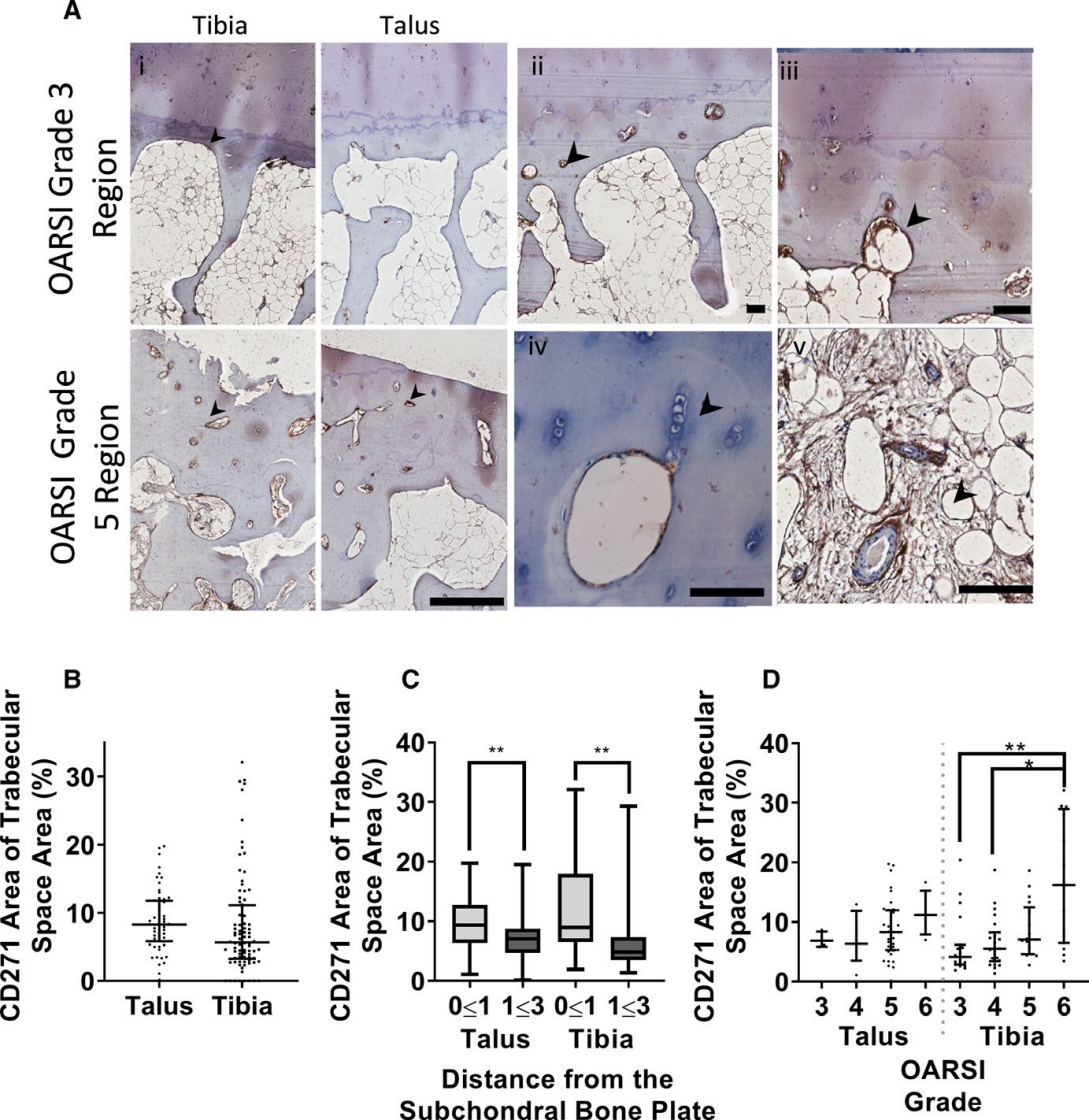
Relationships between CD271‐positive cells and bone behaviour (A) High magnification images of immunohistochemistry for CD271, a native marker of MSCs, showing staining: (i) Overview of grade 3 and grade 5 regions from talus and tibia. (ii/iii) High staining in SB areas beneath the tidemark. (iv) Areas of chondrocyte clustering. (v) bone marrow stroma around blood vessels. B, Comparison of total CD271 coverage between talus and tibia. C, Relative CD271 coverage comparing regions near the subchondral bone plate to that further away. Box plots represent medians and IQR from 3 donors measured from a minimum of 4 individual tiles. D, The effect of overlying cartilage damage grade with CD271 coverage of trabecular space underneath. Symbols on B and D show individual measurements; lines and error bars represent medians and IQR. *P .05, **.01, Kruskal‐Wallis test with Dunn's correction for multiple comparisons

To investigate whether subchondral bone MSCs could be responsible for forming new bone (and not cartilage) in ankle OA talus and distal tibia, immunohistochemistry for CD271+ cells was next performed. In the areas with minimal cartilage damage (grade 3), CD271+ staining was found on the cells lining trabeculae, as expected (i, top panels).^25^ Comparing less and more damaged regions (Figure 5Ai top vs bottom panels), CD271+ staining increased in worse‐damaged (grade 5) regions, where positive staining increased particularly near the tidemark, within small cavities of the thickened SBP and lining trabecular spaces (Figure 5Aii‐iii). Interestingly, CD271+ staining was identified next to proliferating chondrocytes in deep cartilage areas (iv), as well as prominent in marrow spaces with stromal fibrosis (v).

To quantify MSC abundance, whole sample mosaics up to 3 mm depth were created using stitched tiles, with each tile individually analysed for CD271+ area as a fraction of trabecular space area. In general, the talus and tibia showed similar abundance of CD271+ staining, if slightly higher in the talus (8.3% in talus vs 5.6% in tibia (Figure 5B)). Subsequent comparisons of CD271 coverage with depth showed that CD271+ cells were most frequent near the SBP, with ~10% coverage in the first millimetre, falling significantly to 4% (*P* = .0031) and 6% (0.0028) in the talus and tibia, respectively, at 1‐3 mm depth compared with 0‐1 mm depth (Figure 5C). There was a clear association between the grade of cartilage damage and the amount of CD271 staining within trabecular spaces, with significant differences in the tibia between grade 3 and 6 (*P* = .008), and 4 and 6 (*P* = .0197) (Figure 5D). This association is consistent with MSC behaviour primed for bone synthesis in the areas of cartilage damage and consequently, regions with abnormal biomechanics, particularly in the tibia.

## 4 DISCUSSION

Many treatments of ankle OA, as well as osteochondral lesions of the talus, rely upon endogenous cells for repair, including microfracture and joint distraction, among others.^41^ However, the quality and quantity of endogenous repair cells facilitating these treatments, including MSCs themselves, remain under‐investigated.

In the present study, we show for the first‐time subchondral bone MSC presence in both OA talus and tibia, with both talar and tibial MSCs showing enhanced chondrogenesis and osteogenesis than control IC MSCs, and reduced adipogenesis in talar MSCs. MSCs identified by the CD271 marker concentrated in regions of cartilage damage and new bone formation, illustrating an aberrant repair attempt, as seen in other human joints or animal models of OA.^25^, ^28^, ^42^ This indicates that multipotential cells are present in OA, with potential to augment repair enhancing cell homing, chondrogenesis for cartilage repair and inhibiting osteogenesis, rather than injecting exogenous MSCs. By analysing both healthy and OA ankle tissues using 3D and 2D methods, the present study confirmed that bone changes in the ankle were similar to that in the inverted knee, with thickening of the SBP, and increased density of bone near the joint surface, with the talus matching changes to the femur, and distal tibia to the proximal tibia.^43^

Analysis of the mCT data for non‐diseased specimens revealed that the SBP was thicker in tibia than the talus, and surprisingly, the overall BV/TV trend was the opposite, with a higher talar BV/TV in non‐diseased state than tibia. Depth‐dependent BV/TV correlated inversely with distance from the SBP, with the highest BV/TV at the joint surface. Previous work has shown that cartilage in the talus and distal tibia has similar thickness and mechanical properties.^44^, ^45^ Therefore, the main reason for this trend would appear to be the loading profile, with the convex talus directing force into the centre of the arch, whereas the concave tibia will have force over the whole SBP. In the knee, the convex femur has a thinner SBP, and the concave tibial plateau has a thicker SBP, showing the same trend.^46^ As the shape of the bones converges force at the SBP or the centre of the arch, underlying bone mitigates less force, explaining this trend and emphasizing the role of biomechanical forces in normal bone structure of the ankle bones.

Comparing non‐diseased to OA tissue, the SBP of the OA‐affected talus was thicker than the tibia, opposite than in non‐diseased specimens. SBP thickening is demonstrated in OA of both ankle and other joints, so the fact that the talus thickens more so than tibia is more likely to be due to local biomechanical differences, which may determine functional responses from endogenous cells including MSCs.^24^, ^47^ Overall BV/TV did not change in OA, but depth‐dependent analysis revealed higher BV/TV in the first 1 mm than healthy, and lower BV/TV after 2 mm, more drastically in the talus than the tibia. Previous research shows BV/TV changes are both joint and depth specific. The hip demonstrates increases in overall BV/TV in OA, but like the present study the knee does not.^43^, ^47^ Segmentation by depth showed BV/TV in the OA knee is always higher than non‐diseased tissue, suggesting different biomechanical OA‐mediated changes between knee and ankle.^43^, ^48^, ^49^ As explained earlier, this is likely due to the loading profile of the two talocrural bones relative to other joints.^50^ Additionally, the talocrural joint is extremely congruent relative to other bones, which may also explain the differences to the knee.^50^ The present data demonstrate that OA structural changes occur mostly at the joint surface. SBP thickness increased more so in the talus, conversely BV/TV changes were greater in the tibia, suggesting different biomechanical adaptation to resist OA. It is possible that cartilage loss results in higher forces for the bone to mitigate, and so bone is synthesized at the naturally thinner SBP of the talus, whereas in the tibia as the plate is already thick, the relatively thinner bone just below will thicken.

Next, subchondral bone MSC presence and topography in relation to osteochondral changes in both distal tibia and talus was investigated. Li et al^51^ showed MSC presence in the calcaneus, another bone of the ankle, although by bone marrow aspiration, compared with this study isolating a higher proportion of MSCs by using collagenase bone digest. Isolated OA talar and tibial cells presented typical MSC surface phenotype, trilineage differentiation capacity and plastic adherence, the main criteria forth set by the ISCT.^33^ Similar to OA hip or knee, the relative frequency of OA MSCs was much higher than found in the IC.^25^, ^28^ Both distal tibial and talar MSCs showed higher GAG synthesis than iliac crest, showing a higher affinity for cartilage synthesis. Differentiated talar MSCs trended higher calcium deposition and significantly lower adipogenesis than the IC, whereas tibia did not. Lower talar MSC adipogenesis than tibia may be due to the talus having a higher BV/TV, meaning less trabecular cavity space and less need for fat as a space filler and load distributor.^8^ The present study demonstrates that endogenous MSCs of both the talus and tibia have the capacity for repair. The reduced inclination for adipogenesis may drive the improved outcome of microfracture in the ankle relative to other joints.^52^, ^53^ However, other factors such as unique ankle biomechanics and donor age may play a role.^44^, ^45^ As ankle OA patients are usually younger, this enhanced repair capability may have also been driven by these less aged MSCs, as MSCs are known to lose differentiation capability in older patients.^54^ As iliac crest and ankle tissues were not precisely age matched in the present study, the fact that talocrural chondrogenic and osteogenic MSC differentiation appeared more potent than the relatively younger iliac crest MSCs furthers the argument that talocrural MSCs have a greater ability to initiate osteochondral joint repair. The higher GAG synthesis in both talar and tibial MSCs than iliac crest suggests a higher inclination for cartilage repair; however, it would be interesting in future to investigate the level of type I or type II collagen synthesis to assess whether synthesis favours fibrocartilage (predominantly type I collagen) or hyaline cartilage (type II), which may help to explain why microfracture only appears to work in the short term.^55^ This could be done by both transcript and protein levels.

The talus and tibia both had similar levels of typical OA damage, and presented typical hallmarks as found in the hip or knee.^37^ OARSI scores between both the talus and tibia were similar, although slightly higher in the talus, akin to that of similar research.^56^ Curiously, BA/TA was significantly correlated with OARSI grade in tibia, but not in talus. As the talus had twofold higher BA/TA in grade 3 tissue than tibia (Figure 4D), this may mean the talus resists increases due to a bone density limit. In previous research of the OA knee, the highest medial condyle BA/TA was 60% higher than the OA lateral condyle, similar to the upper range of the talus in this study.^25^ As shown by mCT in the present study, SBP thickness was higher in OA tissue than non‐diseased, and BA/TA increased most in the first millimetre from the SBP. The SBP appears to compensate for cartilage destruction, focusing forces at the joint surface. Micro‐cracks in the SBP due to OA may also be enabling factors in synovial fluid and cartilage to reach bone. This crosstalk may be compounding thickening of the bone further at superficial regions, on top of biomechanical stimuli.^57^, ^58^

As a marker of bone‐resident MSCs, CD271 has previously been used to look at changes in MSC localization in OA.^26^, ^27^ In this study, talus showed slightly higher overall CD271 staining than tibia, potentially correlating with the increased SBP thickening in talus. Similar to BV/TV changes, significantly more CD271+ MSCs were found within the first millimetre from the SBP compared with the next two millimetres in both bones. Additionally, CD271 area increased with OARSI damage grade particularly in the tibia consistent with greater BV/TV rise in response to cartilage damages in the tibia compared with talus. As such it appears, MSCs are involved in bone synthesis, with picrosirius red (Figure S1) showing where bone has thickened, that it is indeed new bone. Progressive bone formation in talocrural subchondral bone was also evident by co‐localization of osteoblast progenitors, cuboid osteoblasts and early osteocytes to the same outer areas of trabeculae(Figure S2). As such, in vivo it appears MSCs may be key drivers of OA bone sclerosis, and correcting their behaviour may slow or prevent OA progression. Gene expression profiling of uncultured talocrural MSCs, as performed using knee MSCs from more and less damaged regions,^25^ would be necessary to confirm MSC redisposition to osteogenesis in OA talocrural tissues.

This study is limited by small numbers of ankle OA patients and the amount of tissue that could be removed during surgery, which aimed to minimize harm to the patient. Additionally, the choice of iliac crest as a control issue is a limitation, as this is not an articulating joint region, with no need for cartilage synthesis and much less biomechanical stimuli. Although ideally non‐diseased talocrural tissue would have been used, such samples are ethically complex, and the commercially available frozen cadaveric tissue was not suitable for functional MSC work. Iliac crest MSCs, as the best studied MSC type, are commonly used as a comparator in many previous studies and in our previous work, no gross differences in osteo‐ and chondrogenesis were observed between hip OA and IC MSC^36^, ^39^ suggesting that the present findings showing high osteo‐ and chondrogenesis in talocrural OA MSCs may be ankle joint‐specific, compared with IC. More consideration for age matching of talocrural and control tissues should be given in the future, considering MSC chondrogenesis in particular, declines with donor age.^59^ Due to the small amounts of tissue, particularly for the talus, we were unable to perform flow cytometry for the CD271 marker to quantify MSCs in talocrural tissues and compare their numbers with CFU‐F numbers, or correlate with in situ staining data, as we performed in our other works.^25^, ^28^, ^29^, ^39^ Future studies using sorted CD271+ MSCs from talocrural bone followed by gene expression and functional analysis of the sorted cells would be needed to shed more light on potentially differential responses of talar and tibial MSCs to the OA process in the ankle, and guide towards more region‐specific therapies for ankle OA.

To logically design treatments to reverse or slow down OA progression, first we need to understand disease progression, and why natural repair processes are failing. This study shows similar abundance and in vitro potencies of MSCs in OA‐affected talus and distal tibia. It highlights different biomechanical environments these MSCs are exposed to, both in health and in OA. Our data showing natural resistance of talar MSCs to adipogenesis provides potential explanation for the success of talar microfracture compared with other joints although other factors may be also at play.^13^, ^53^ Our results show that in OA, CD271+ cells associate near the SBP, and accumulate with tissue damage. As these MSCs localize to the joint surface, it is possible that the current technique of drilling until there is a release of fat from the bone, is too deep, and could inform other treatments such as nanofracture, reducing the penetration depth but keeping the benefits of lessened tissue damage.^60^ Focusing on new methods of MSC stimulation to form hyaline cartilage formation over bone synthesis is needed. This work also shows that future work should focus on the modulation of biomechanical environments in different bones in the ankle joint. Some success in this area has been already achieved with ankle joint distraction.^60^, ^61^, ^62^ Combining both biological and mechanical stimulation to direct MSC repair behaviour towards cartilage formation, and predicting how cells respond to a variety of biological and mechanical environments, may lead to new joint‐preserving treatments and avoid major surgical interventions.

## CONFLICTS OF INTEREST

The authors have no conflicts of interest to disclose.

## AUTHOR CONTRIBUTIONS

**William G. Jones:** Conceptualization (equal); Data curation (lead); Formal analysis (equal); Funding acquisition (equal); Investigation (lead); Methodology (lead); Software (equal); Validation (equal); Visualization (equal); Writing‐original draft (equal); Writing‐review & editing (equal). **Jehan J. El‐Jawhari:** Conceptualization (equal); Data curation (equal); Resources (supporting); Supervision (equal); Writing‐review & editing (lead). **Claire L. Brockett:** Conceptualization (equal); Data curation (supporting); Funding acquisition (supporting); Project administration (supporting); Resources (supporting); Supervision (supporting). **Lekha Koria:** Methodology (supporting); Software (supporting); Validation (supporting); Writing‐review & editing (supporting). **Ioannis Ktistakis:** Resources (equal); Writing‐review & editing (supporting). **Elena Jones:** Conceptualization (equal); Data curation (equal); Formal analysis (equal); Funding acquisition (lead); Project administration (lead); Resources (lead); Software (supporting); Supervision (lead); Writing‐original draft (supporting); Writing‐review & editing (lead).

## Supporting information

Fig S1

Fig S2

## Data Availability

The data associated with this paper are available from the corresponding author upon request.
