# Supplementary figures and images for: Multipotential stromal cells in the talus and distal tibia in ankle osteoarthritis – Presence, potency and relationships to subchondral bone changes
Source: J Cell Mol Med. 2020 Dec 11;25(1):259–71. doi: 10.1111/jcmm.15993 (PMC7810934; doi:10.1111/jcmm.15993)

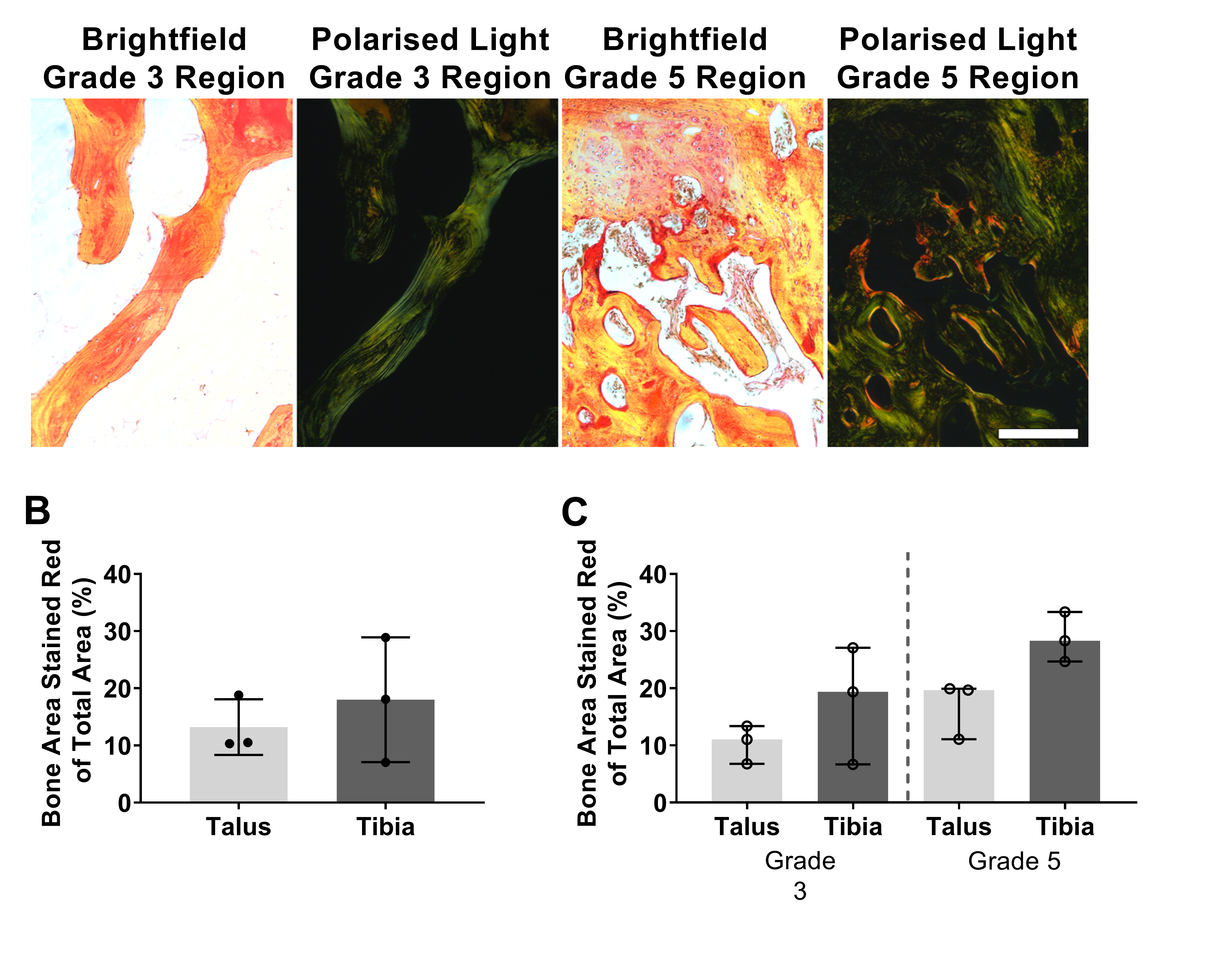

Supplement: Supplementary file 1 — Fig S1 [file JCMM-25-259-s001.tif]

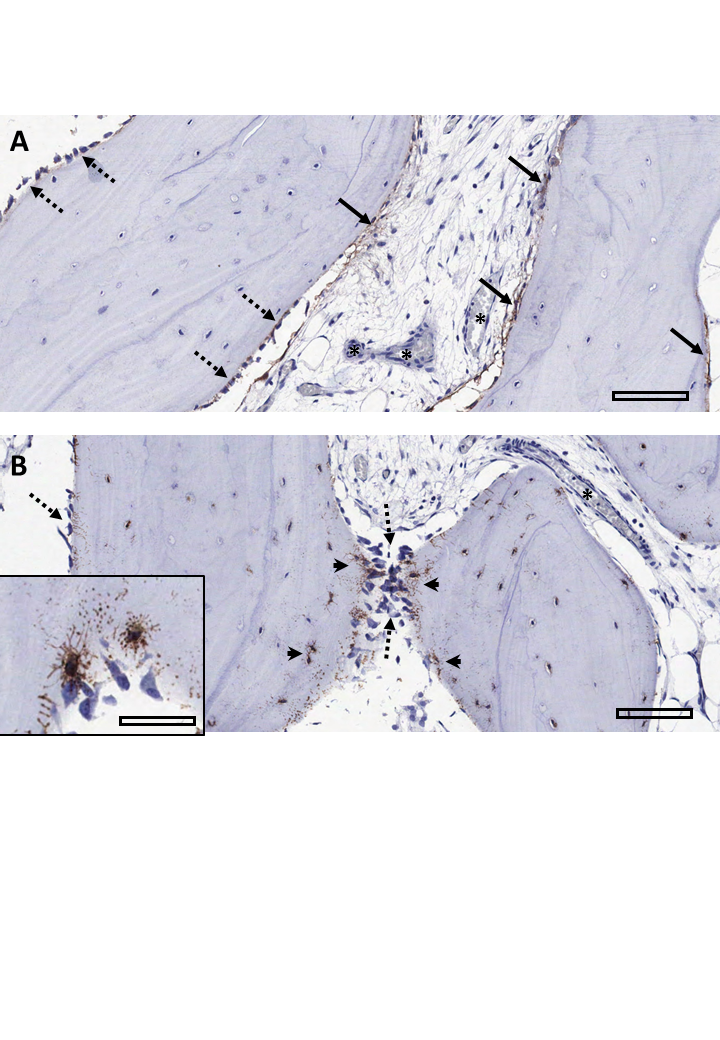

Supplement: Supplementary file 2 — Fig S2 [file JCMM-25-259-s002.tif]
